# *Halophilanema prolata *n. gen., n. sp. (Nematoda: Allantonematidae), a parasite of the intertidal bug, *Saldula laticollis *(Reuter)(Hemiptera: Saldidae) on the Oregon coast

**DOI:** 10.1186/1756-3305-5-24

**Published:** 2012-02-01

**Authors:** George O Poinar

**Affiliations:** 1 Department of Zoology, Oregon State University, Corvallis, OR 97331, USA

**Keywords:** *Halophilanema prolata *n. gen., n. sp., Allantonematidae, Saldidae, intertidal parasite, *Saldula laticollis*

## Abstract

**Background:**

It is rare to find terrestrial nematode lineages parasitizing arthropods inhabiting the intertidal or littoral zone of the oceans. During an ecological study along the Oregon dunes, an allantonematid nematode (Tylenchomorpha: Allantonematidae) was discovered parasitizing the intertidal shore bug, *Saldula laticollis *(Reuter)(Hemiptera: Saldidae). This shore bug is adapted to an intertidal environment and can survive short periods of submergence during high tides. The present study describes the nematode parasite and discusses aspects of its development, ecology and evolution.

**Methods:**

Adults and last instar nymphs of *S. laticollis *(Hemiptera: Saldidae) were collected from the high intertidal zone among clumps of *Juncus *L. (Juncaceae) plants at Waldport, Oregon on October 3, 2011. The bugs were dissected in 1% saline solution and the nematodes killed in 1% Ringers solution and immediately fixed in 5% formalin (at 20°C). Third stage juveniles removed from infected hosts were maintained in 1% saline solution until they matured to the adult stage, molted and mated.

**Results:**

*Halophilanema prolata *n. gen., n. sp. (Nematoda: Allantonematidae) is described from last instar nymphs and adults of the intertidal bug, *Saldula laticollis *on the Oregon coast. The new genus can be distinguished from other genera in the Allantonematidae by a stylet lacking basal knobs in both sexes, an excretory pore located behind the nerve ring, ribbed spicules, a gubernaculum, the absence of a bursa and the elongate-tubular shape of the ovoviviparous parasitic females. Studies of the organogenesis of *Halophilanema *showed development to third stage juveniles in the uterus of parasitic females. Maturation to the free-living adults and mating occurred in the environment. The incidence of infection of *S. laticollis *ranged from 0% to 85% depending on the microhabitat in the intertidal zone.

**Conclusions:**

Based on the habitat and morphological characters, it is proposed that *Halophilanema *adapted a parasitic existence fairly recently, evolutionarily speaking. It was probably a free-living intertidal or shore nematode that fed on microorganisms, especially fungi, in the intertidal habitat and became parasitic after saldids entered the environment. *Halophilanema *represents the first described nematode parasite of an intertidal insect.

## Background

It is rare to find nematodes parasitizing arthropods inhabiting the intertidal or littoral zone of the oceans. This is an extremely harsh environment where the occupants are subjected to wave action, high salinity and open exposure. An exception is the mermithid, *Thaumamermis zealandica *Poinar, Latham & Poulin [1] that parasitizes New Zealand intertidal amphipods. In addition, species of the entomopathogenic nematode *Heterorhabditis *Poinar are thought to have evolved in an intertidal habitat from free-living rhabditids [2]. However, based on our present knowledge, the intertidal environment is an unsuitable habitat for all terrestrial lineages of entomogenous nematodes.

Insects are uncommon in the intertidal habitat and only a small number are able to complete their entire development in this domain. One of these few is the shore bug, *Saldula laticollis *(Reuter), which possesses morphological and physiological attributes that enable it to survive along the open coastline, including the ability to endure short periods of submergence during high tides [3]. Adults and mature nymphs of a population of *S. laticollis *collected along the Oregon coast were discovered to be parasitized by an undescribed allantonematid. The present study describes this nematode and discusses aspects of its development, ecology and evolution.

## Methods

**Figure 1:**
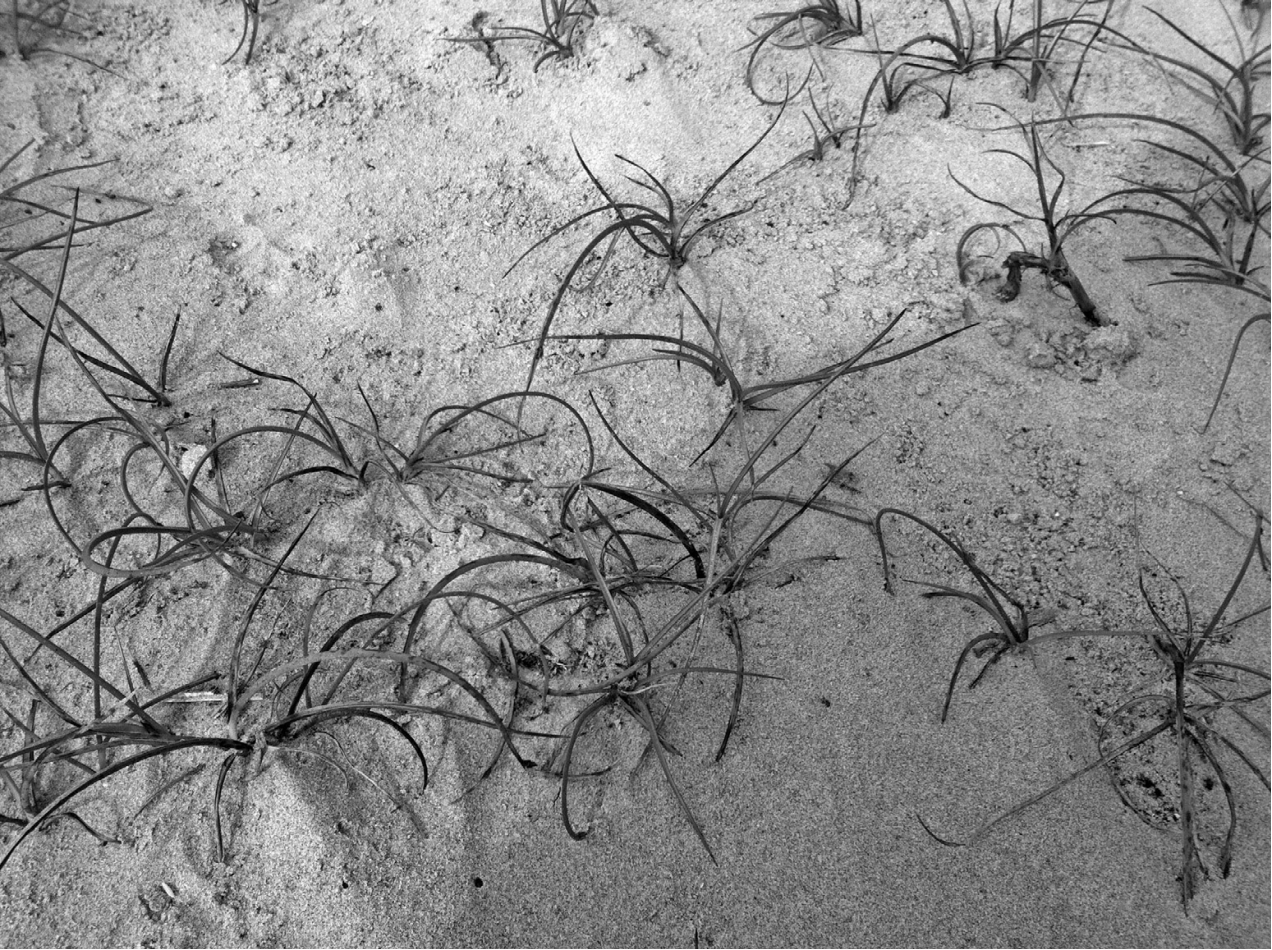
**Upper intertidal habitat of *Saldula laticollis *among partially covered *Juncus *plants**.

Several hundred adults and last instar nymphs of *S. laticollis *(Hemiptera: Saldidae) were collected from the high intertidal zone among clumps of *Juncus *L. (Juncaceae) plants at Waldport, Oregon on October 3, 2011 (Figure 1). The highest concentration of infected bugs was collected at the high tide level in a distinctive area containing yellow-tinted sand. The bugs were dissected in 1% saline solution and the nematodes killed in 1% Ringers solution and immediately fixed in 5% formalin (at 20°C). Measurements were made on fixed nematodes that were processed to glycerin by the evaporation method. Living nematode stages were examined directly to detect fine morphological details and follow their development. Third stage juveniles removed from infected hosts were maintained in 1% saline solution until they matured to the adult stage, molted and mated. The following description is based on parasitic females removed from the host and free-living adults reared from third-stage juveniles taken from 25 infected adult *S. laticollis*. Observations and photographs were made with a Nikon Optiphot Microscope. All measurements, including the average value and those of the range (in parentheses), are in micrometers unless otherwise specified.

## Results and discussion

**Figure 2:**
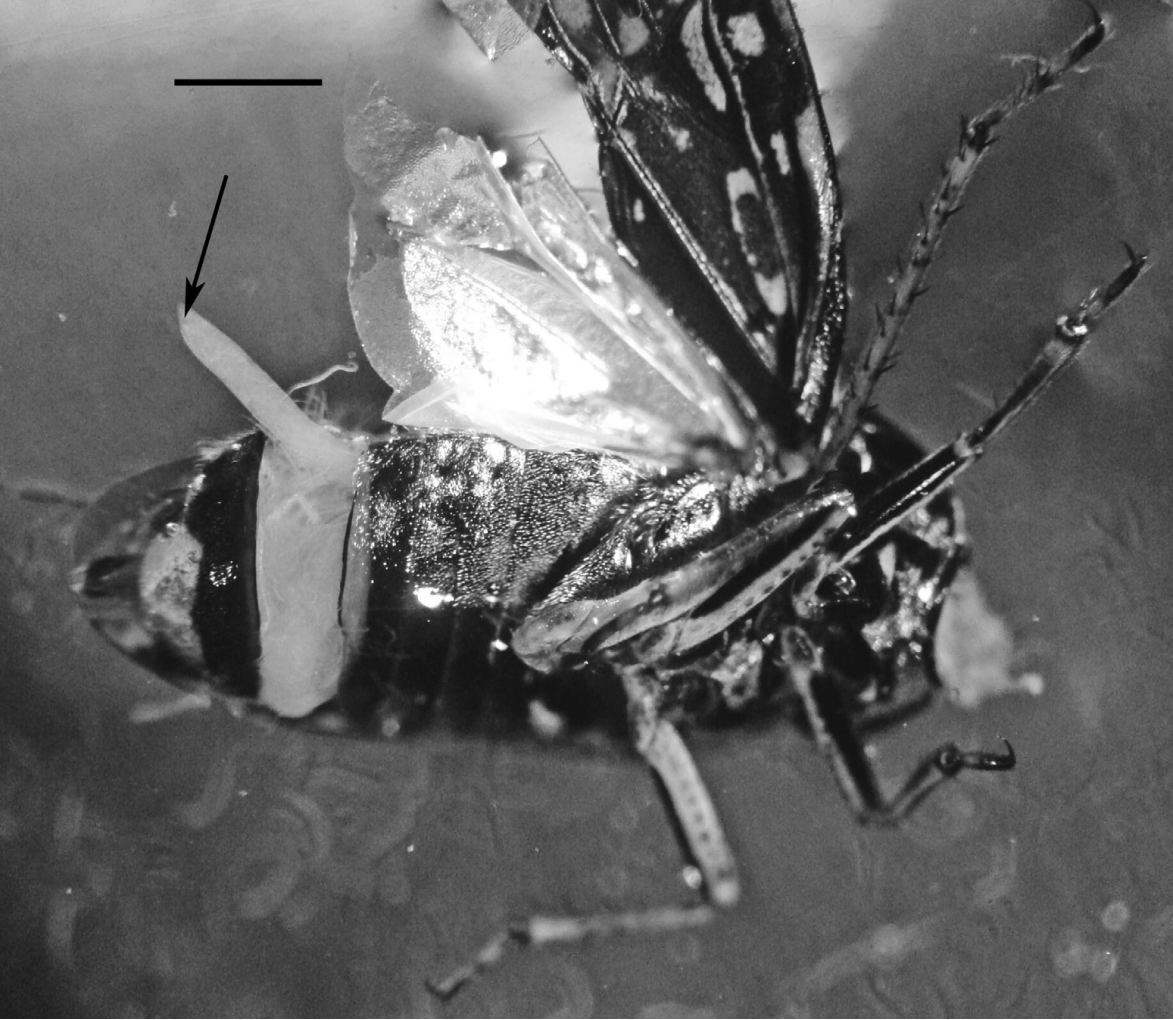
**Parasitic female (arrow) of *Halophilanema prolata *protruding from the abdomen of its host, *Saldula laticollis***. Body cavity of host also contains third stage juveniles that exited from the parasitic female. Bar = 750 μm.

**Figure 3:**
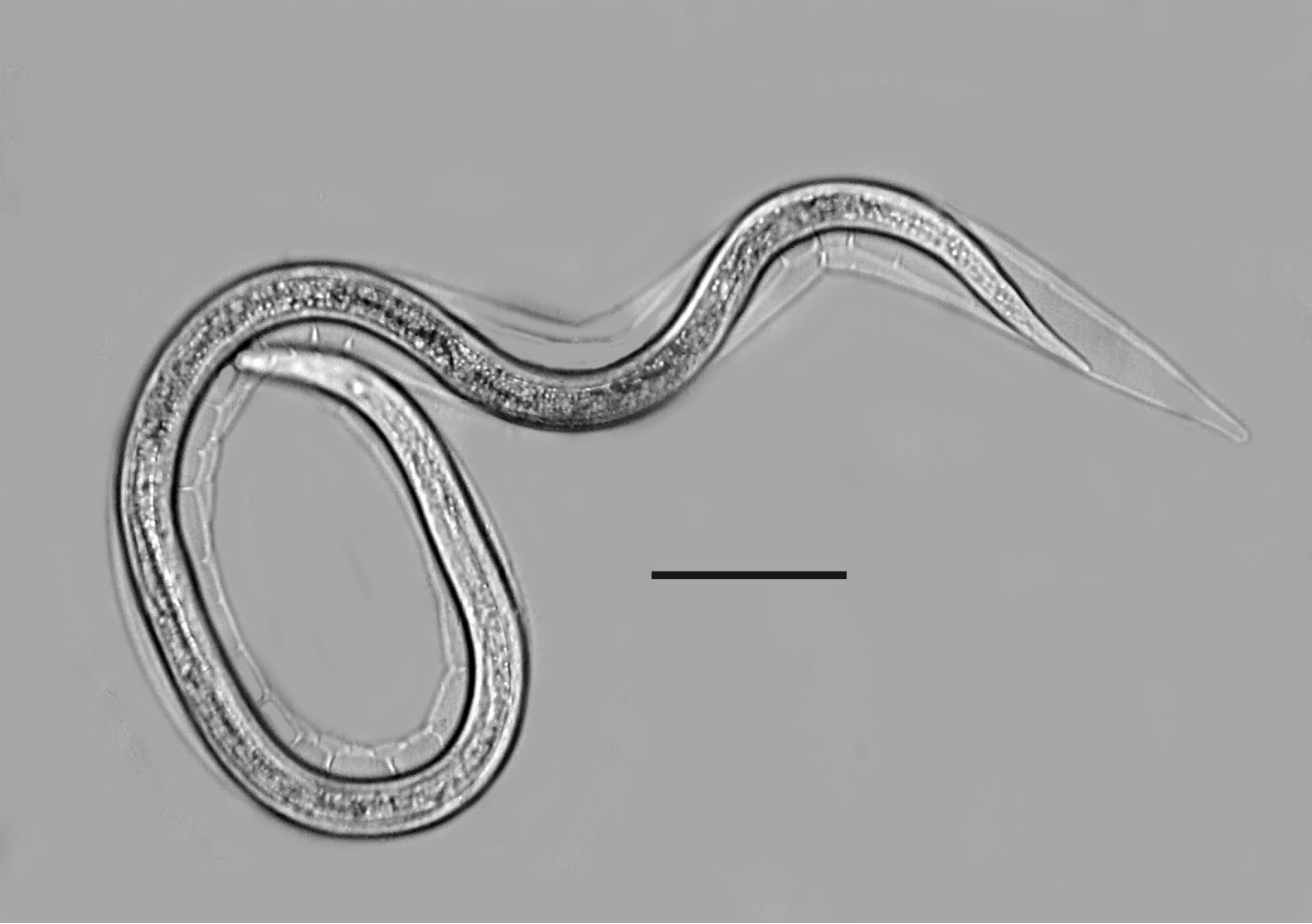
**Double molt of third stage juvenile female of *Halophilanema prolata***. Note that both third and fourth cuticles are being shed simultaneously. Bar = 63 μm.

**Figure 4:**
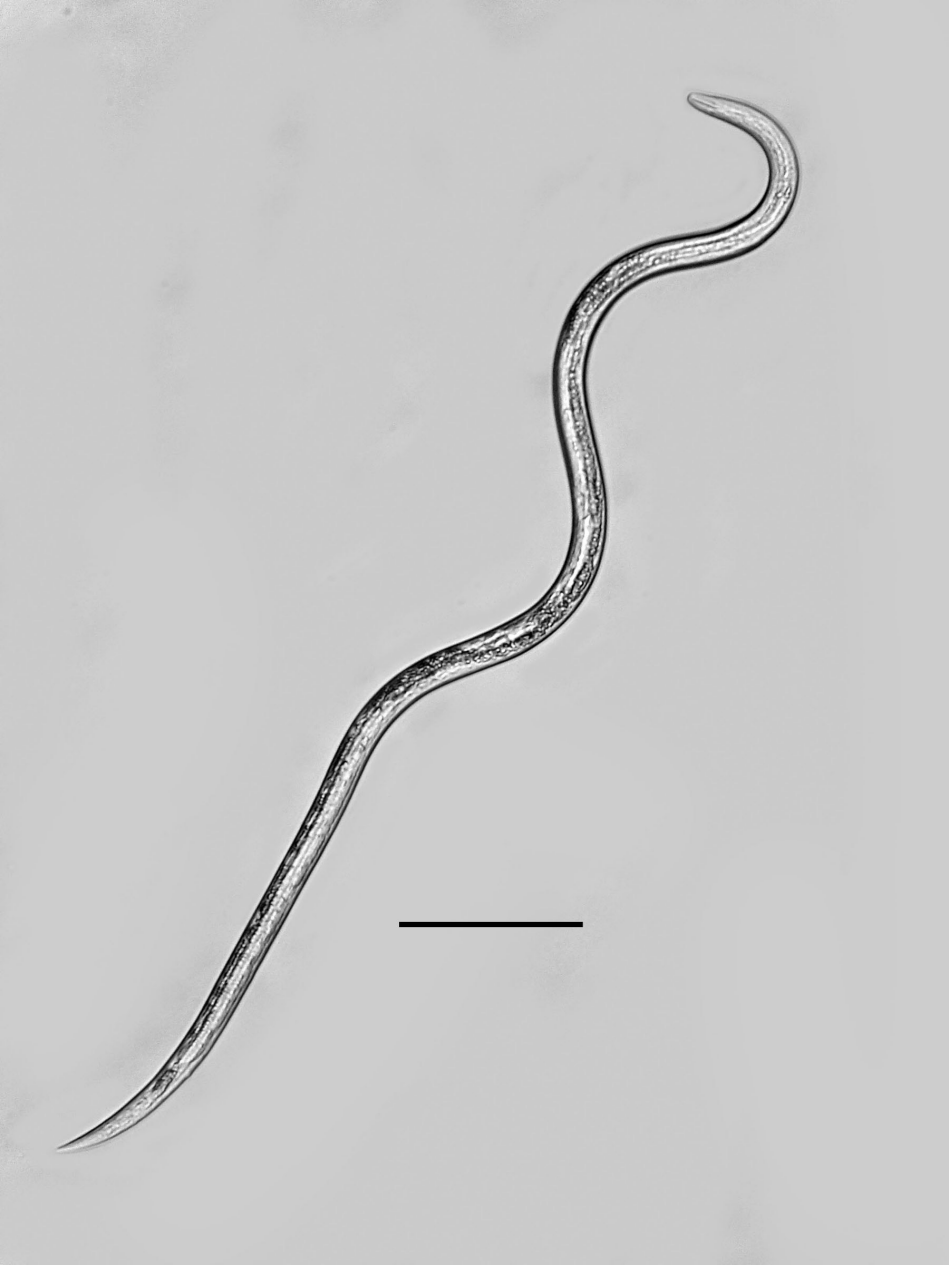
**Free-living female of *Halophilanema prolata *that has shed the third and fourth cuticles**. Bar = 90 μm.

**Figure 5:**
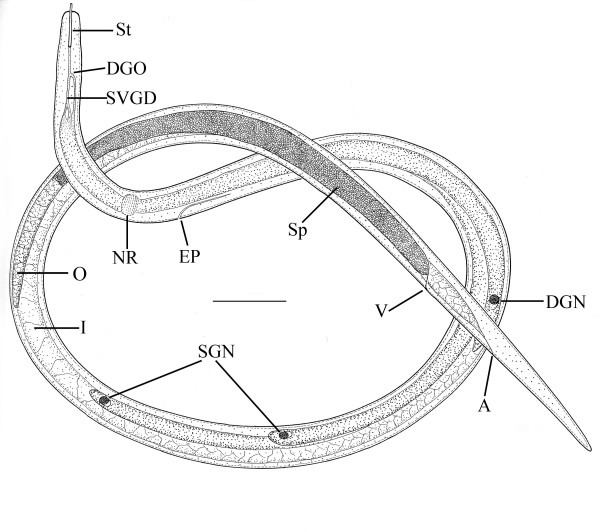
**Free-living female of *Halophilanema prolata***. A = anus; DGN = dorsal gland nucleus; DGO = dorsal gland opening; EP = excretory pore; I = intestine; NR = Nerve ring; O = ovary; SVGD = subventral gland duct; SGN = subventral gland nuclei; Sp = sperm in uterus; St = stylet; V = vulva. Bar = 25 μm.

The yellow-tinted sand at the high tide level where the highest concentration of infected *S. laticollis *occurred contained a mixture of bacteria, algae, fungi, protozoa and free-living nematodes, including stages of the allantonematid parasite. The body cavity of infected *S. laticollis *contained mature parasitic female nematodes and numerous third stage juveniles (Figure 2). Third stage juvenile nematodes removed from the body cavity of *S. laticollis *and held in 1% saline solution molted twice (usually simultaneously) to the adult stage in 5-7 days at 20°C (Figure 3). Mating occurred in the saline solution as soon as the cuticles were shed (Figures 4, 5). Juveniles and fertilized females also were found in the yellow-tinted sand.

### Description of nematode

Tylenchida Thorne, 1949

Sphaerularioidea Lubbock, 1861

Allantonematidae Pereira, 1931

*Halophilanema *Poinar, n. gen.

Type species: *Halophilanema prolata *Poinar

Description: Free-living stages slender with conical-pointed tail; well-developed stylet lacking basal knobs present in both females and males; excretory pore located behind nerve ring; parasitic females elongate-tubular, ovoviviparous; males with distinct gubernaculum; bursa absent; spicule lamina with two lateral (horizontal) ribs; development to third stage juveniles occurs in uterus of parasitic females; free-living stages adapted to saline intertidal conditions.

Comments: Based on the key to the genera of Allantonematidae presented by Siddiqi (2000) *Halophilanema *keys to couplet 9, which ends with *Pratinema *Chizhov & Sturhan, 1998 and *Protylenchus *Wachek, 1955. However *Pratinema *lacks a gubernaculum, the parasitic female is ventrally curved and the stylet is knobbed. *Protylenchus *is characterized by an obese, cylindrical straight parasitic female and a thick, knobbed stylet. Neither genus contains any members with ribbed spicules. *Halophilanema *cannot be placed in any existing genus in this family. It can be distinguished from all existing genera by the following combination of characters: a stylet lacking knobs in both free-living males and females, an excretory pore located behind the nerve ring, uterus without post-uterine extension, a distinct gubernaculum, no bursa, ribbed spicules and the elongate-tubular shape of the parasitic female.

*Halophilanema prolata *Poinar, n. sp.

**Figure 6:**
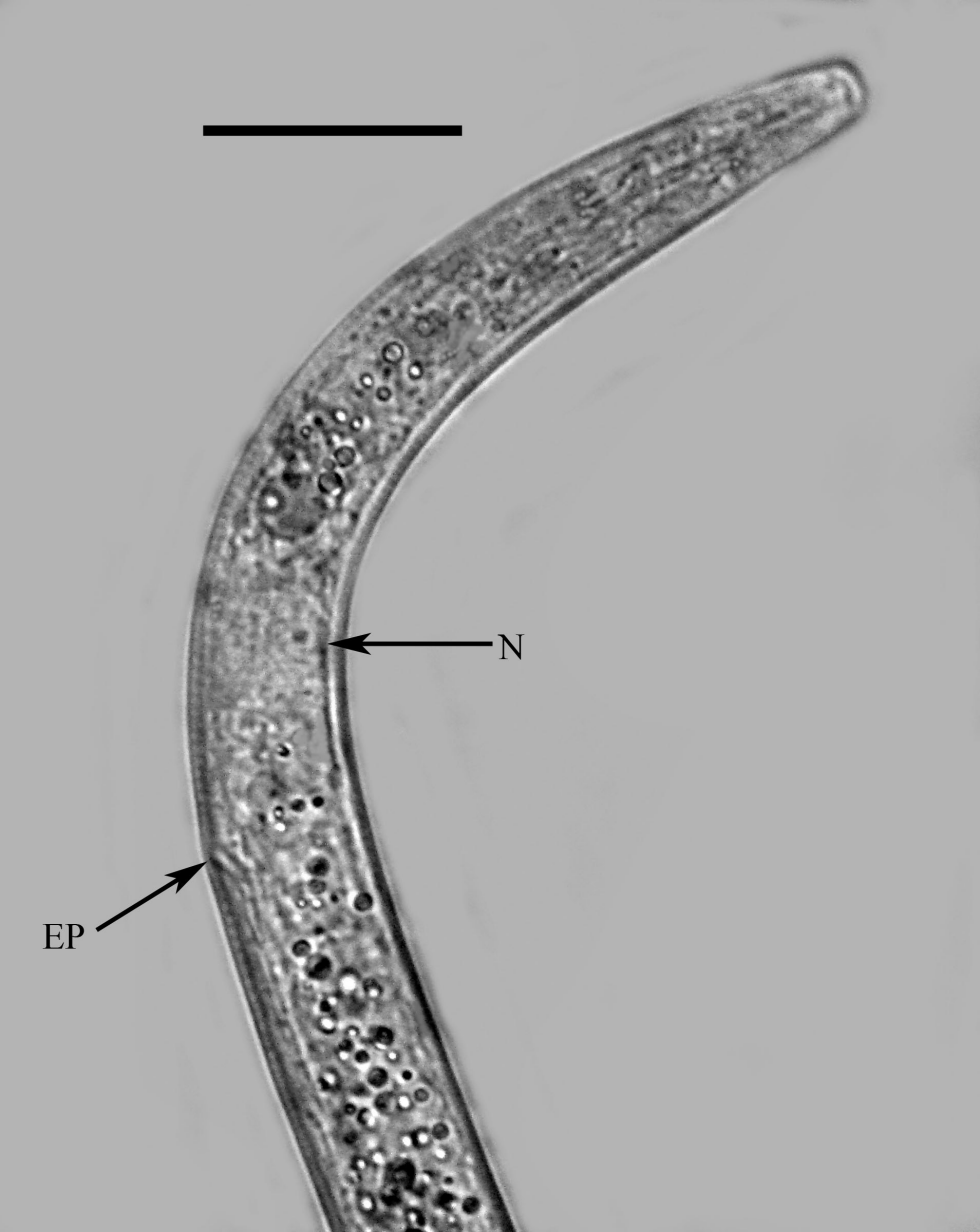
**Head of free-living female of *Halophilanema prolata *showing nerve ring (N) and excretory pore (EP)**. Bar = 25 μm.

Free-living female (n = 12)(Figures 4, 5, 6): total body length, 733 (655-916); greatest body width, 18 (14-27); stylet length, 17 (13-19); head tip with 4 cephalic papillae; head to dorsal gland outlet, 20 (18-29); head to ventral gland outlet, 30 (26-41); head to nerve ring, 75 (65-82); head to excretory pore, 90 (83-98); tail length, 49 (45-51); distance vulva to tail tip, 76 (68-81); % vulva, 90 (88-95).

Body elongate; cuticle smooth; lateral fields 2.5 wide, with 4 incisures; stylet distinct, lacking knobs; dorsal pharyngeal gland opening slightly less than one stylet length behind stylet base; subventral gland openings located approximately 1.5 stylet lengths behind head; pharyngeal glands long, subventral gland sometimes reaching anterior tip of ovary in fertilized females; excretory pore located behind nerve ring; vulva and anal openings faint; uterus without post-uterine extension; ovary outstretched; tail conoid, tip sometimes slightly angular.

**Figure 7:**
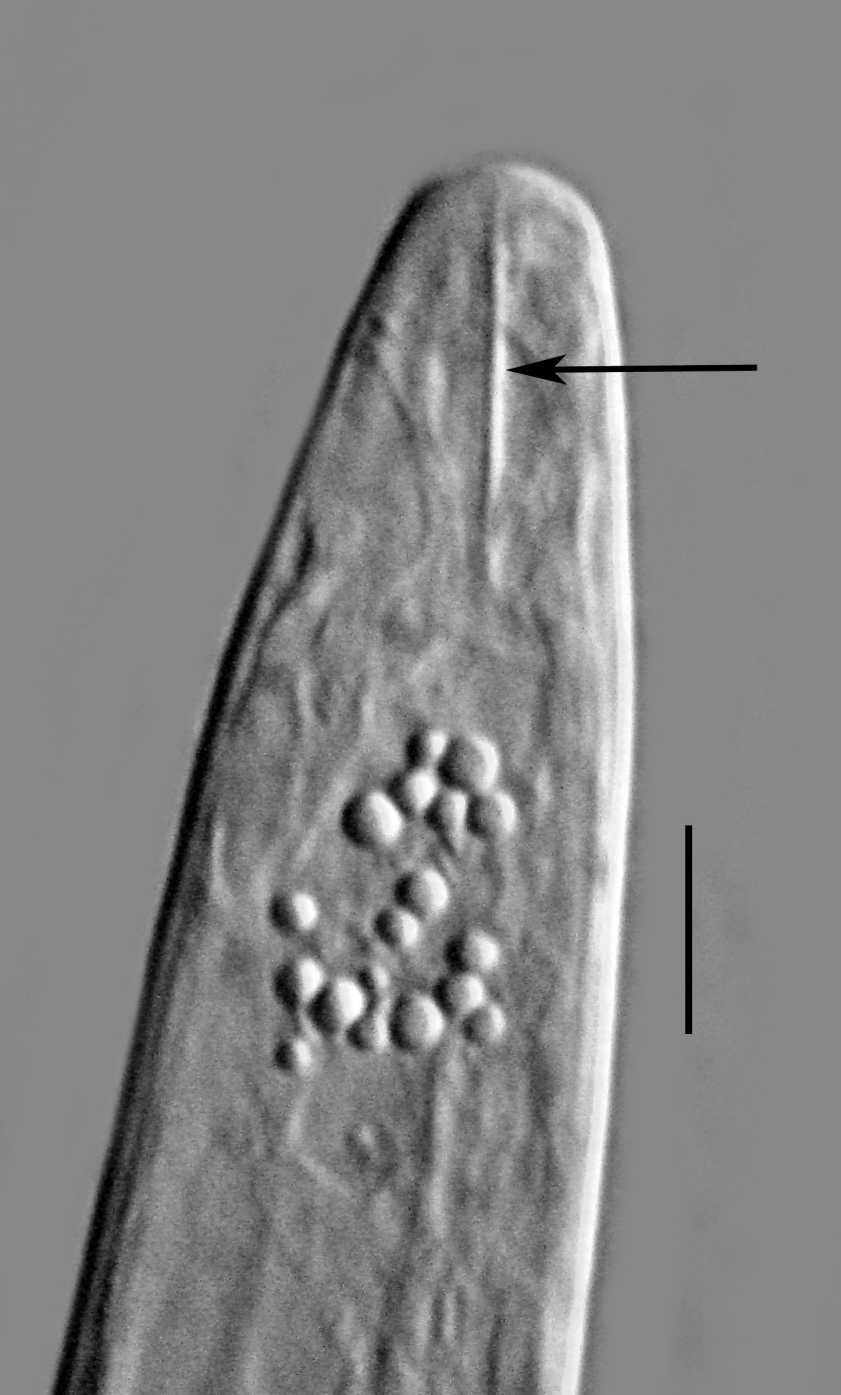
**Stylet (arrow) of male *Halophilanema prolata***. Bar = 5 μm.

**Figure 8:**
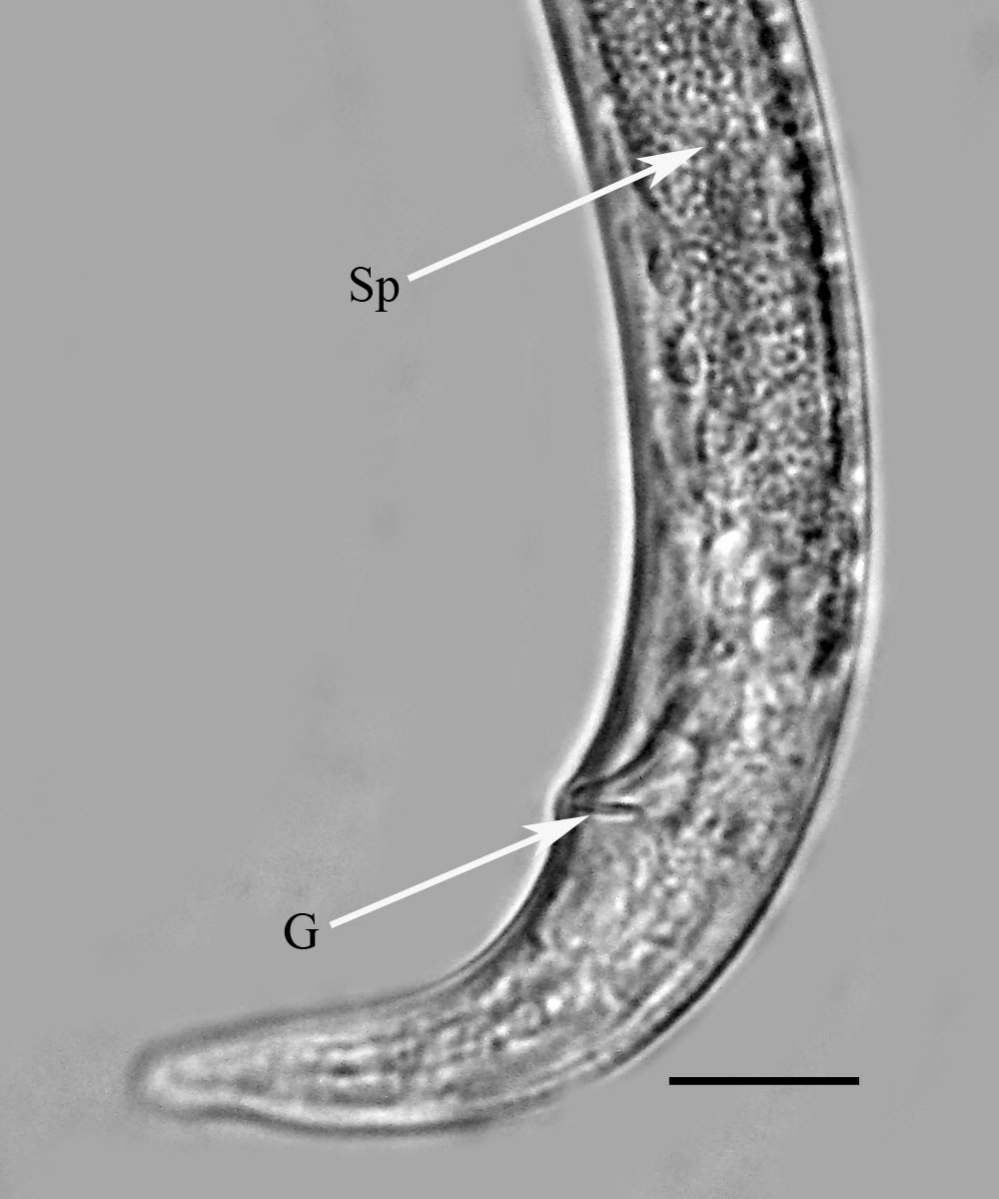
**Male of *Halophilanema prolata *showing gubernaculum (G) and sperm (Sp)**. Bar = 10 μm.

**Figure 9:**
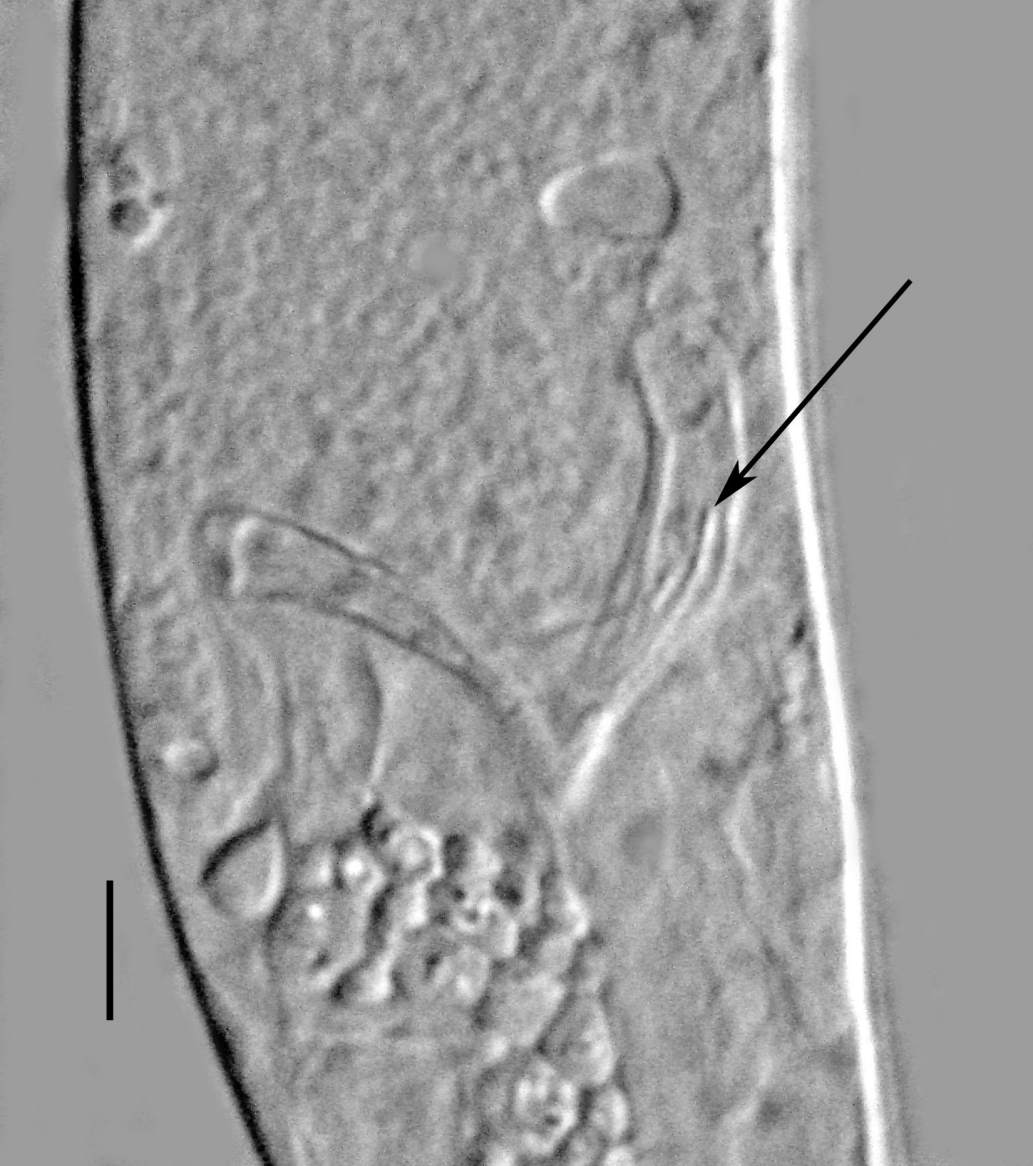
**Ribs on spicule of *Halophilanema prolata***. Arrow shows longer dorsal (lower) rib below shorter ventral (upper) rib. Bar = 4 μm.

Free-living male (Figures 7, 8, 9) (n = 12): total body length, 642 (604-668); greatest body width, 17 (14-21); stylet length, 7 (6-9); head to nerve ring, 39 (37-42); head to excretory pore, 47 (45-50); tail length, 32 (30-34); spicule length, 13 (12-15); spicule width, 5 (4-5); gubernaculum length, 4 (4-5).

Body and stylet shorter than that of female; cuticle smooth, with lateral fields similar to those of female; dorsal and ventral gland outlets present, but glands atrophied; testis outstretched or reflexed; seminal vesicle filled with sperm; spicules separate, slightly cephalated, with thin velum, moderately curved in lateral view, lamina bearing two horizontal ribs; ventral (upper) rib shorter than dorsal (lower); gubernaculum short, prominent, straight, basal portion bearing lateral flanges; bursa absent; tail tip conoid with blunt tip.

**Figure 10:**
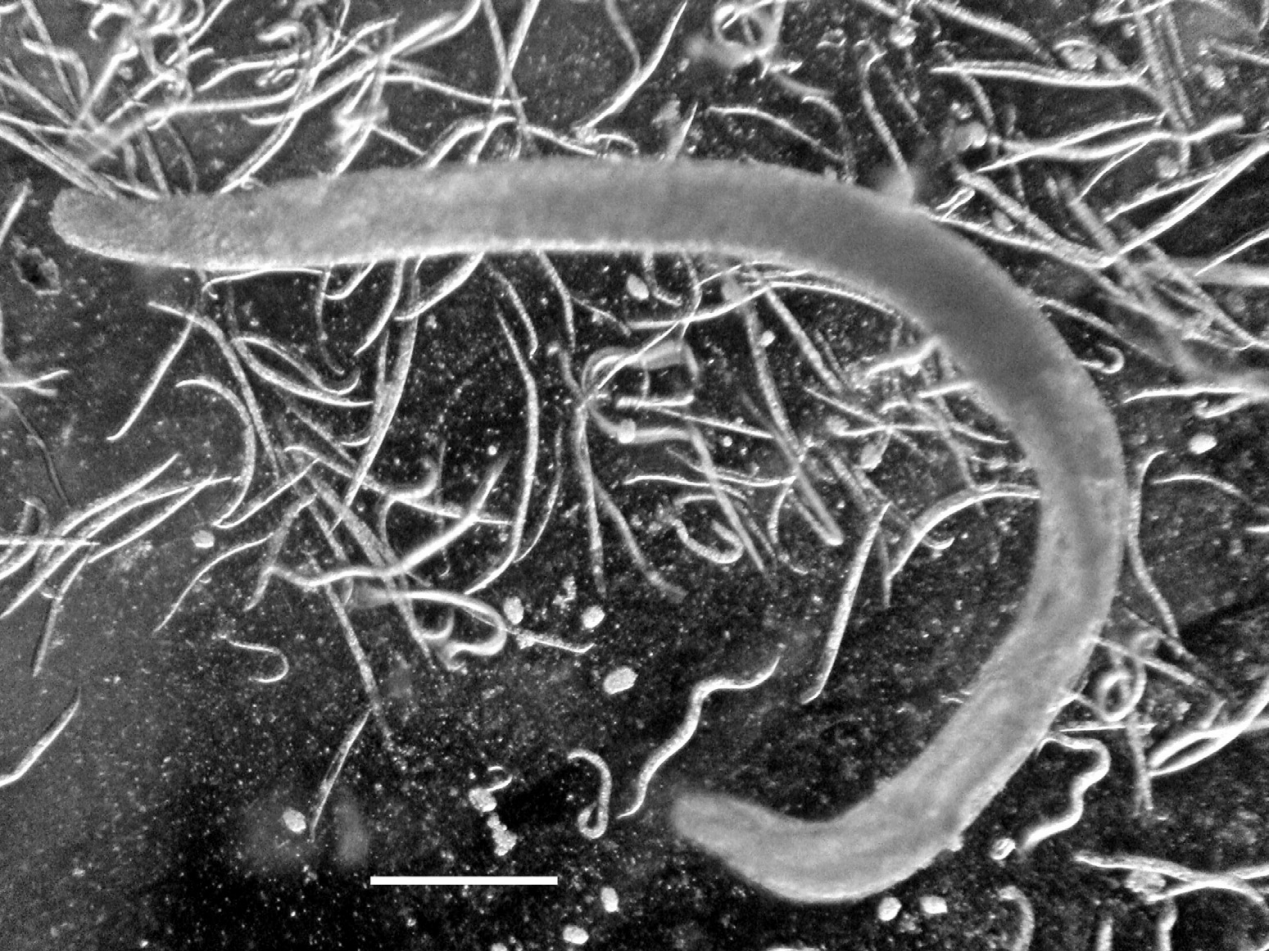
**Parasitic female and immature stages of *Halophilanema prolata***. Bar = 725 μm.

**Figure 11:**
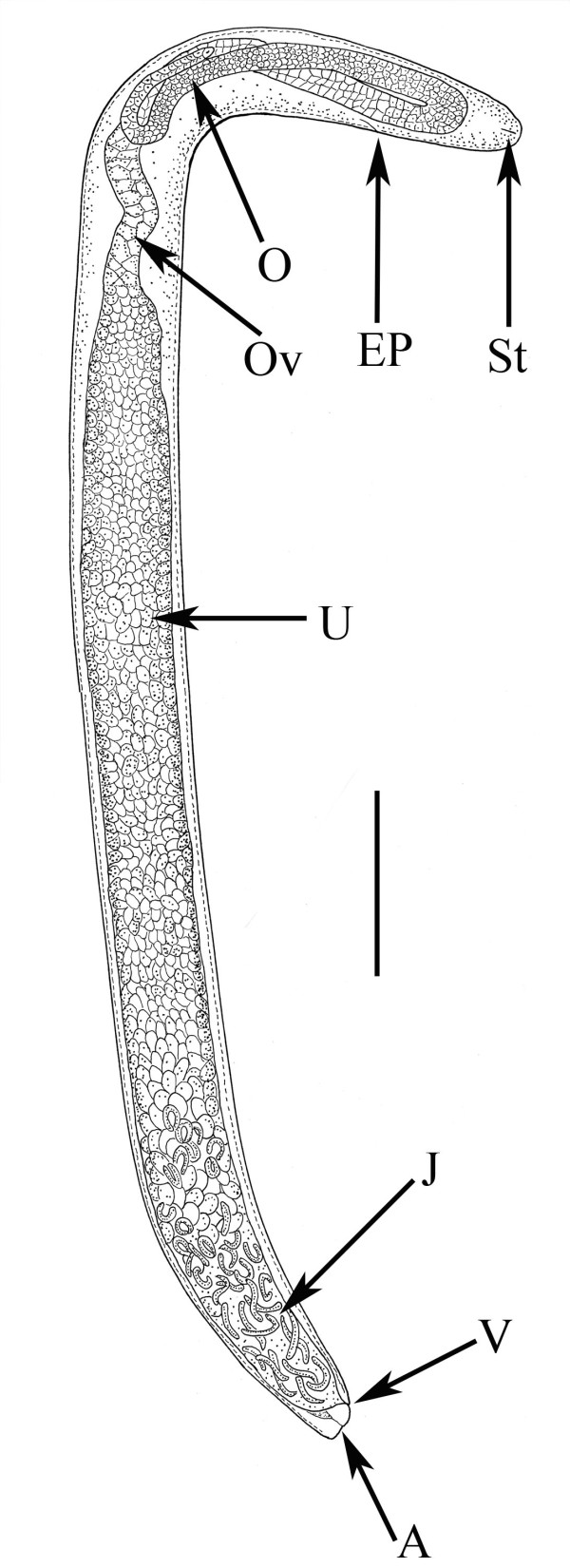
**Parasitic female of *Halophilanema prolata***. A = anus; EP = excretory pore; J = juveniles in lower portion of uterus; O = ovary; Ov = oviduct; St = stylet; U = uterus with developing eggs; V = vulva. Bar = 307 μm.

**Figure 12:**
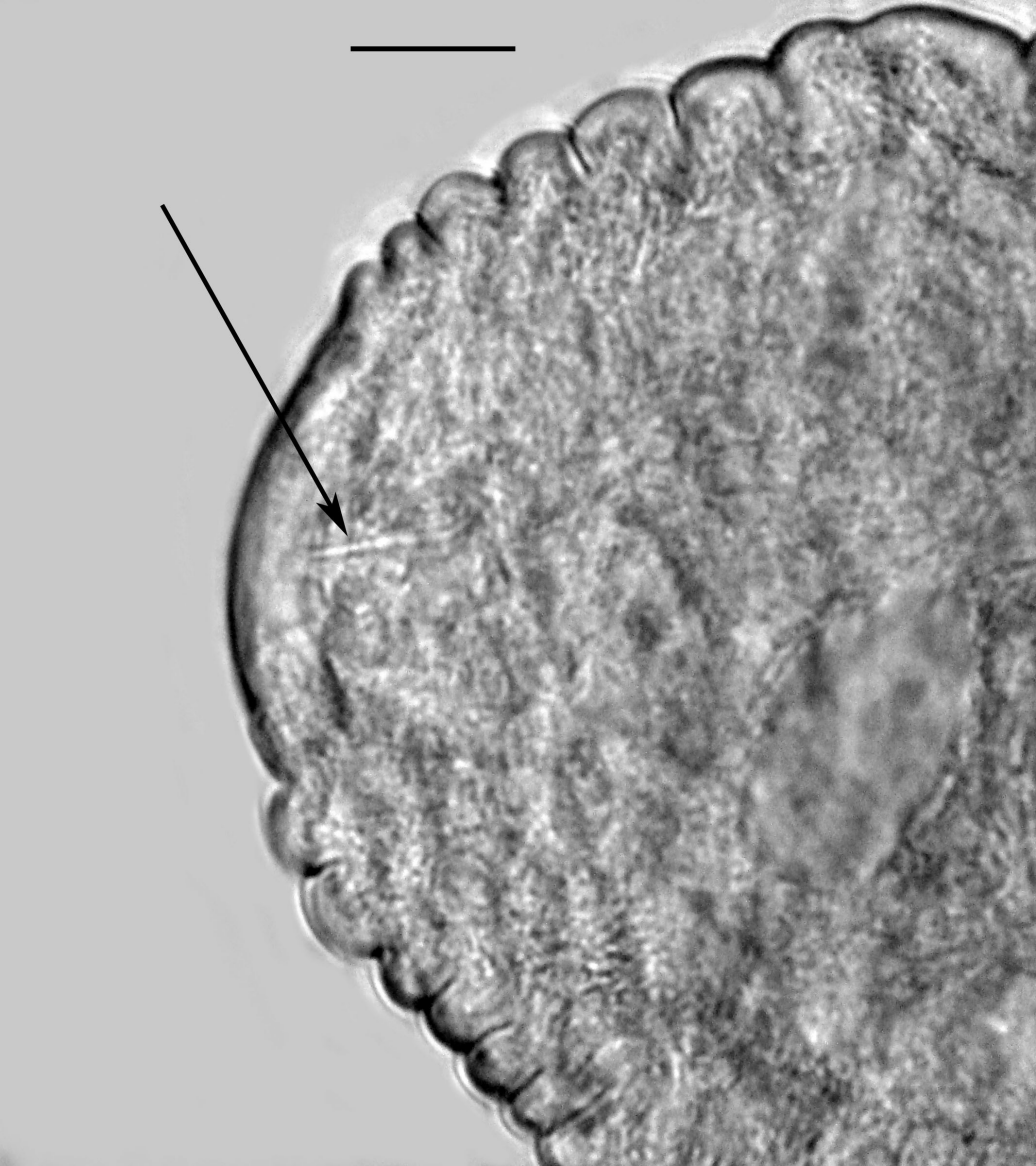
**Stylet (arrow) of *Halophilanema prolata *parasitic female**. Bar = 18 μm.

**Figure 13:**
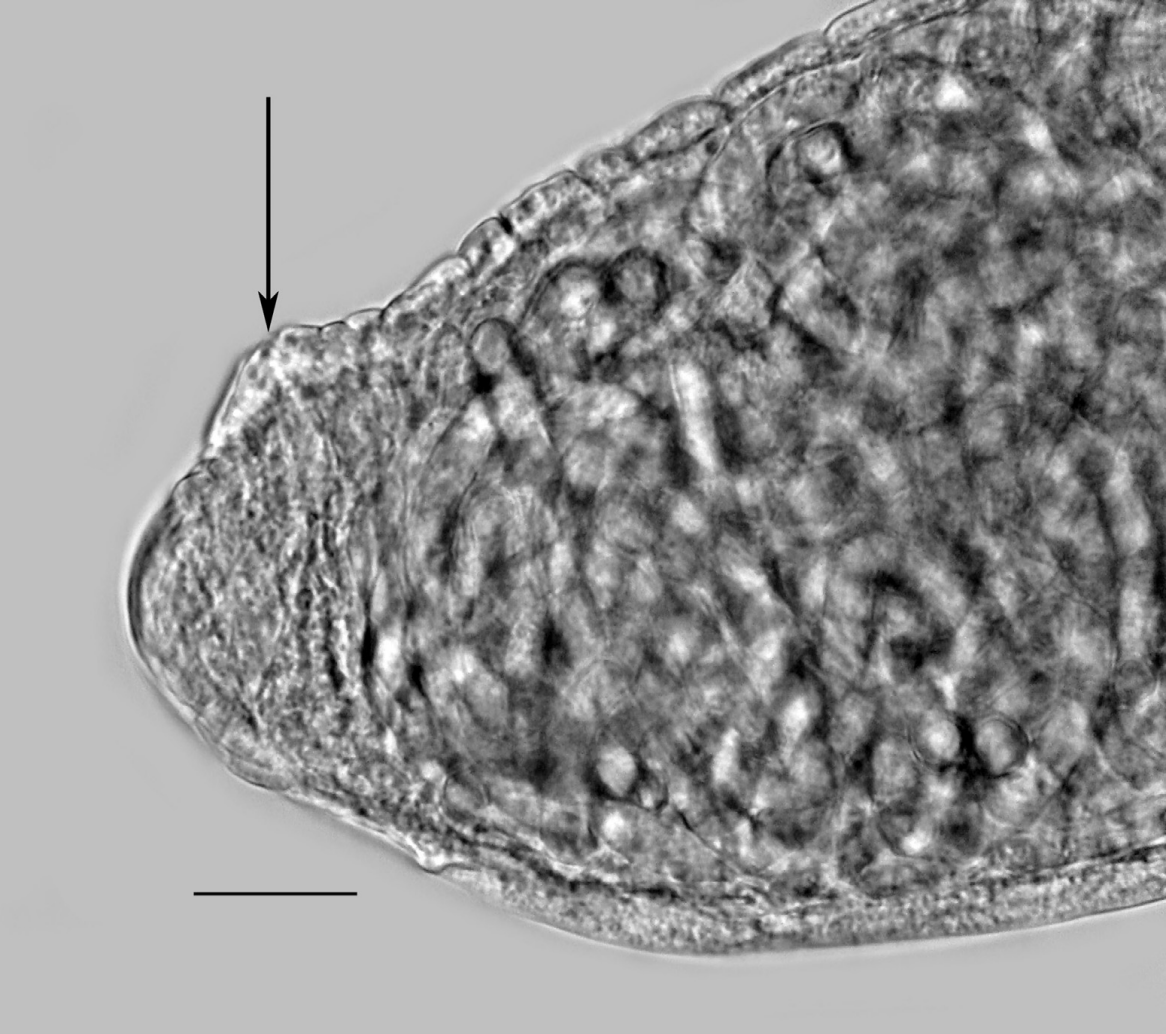
**Subterminal vulva (arrow) of *Halophilanema prolata *parasitic female positioned on a short prominence**. Bar = 51 μm.

**Figure 14:**
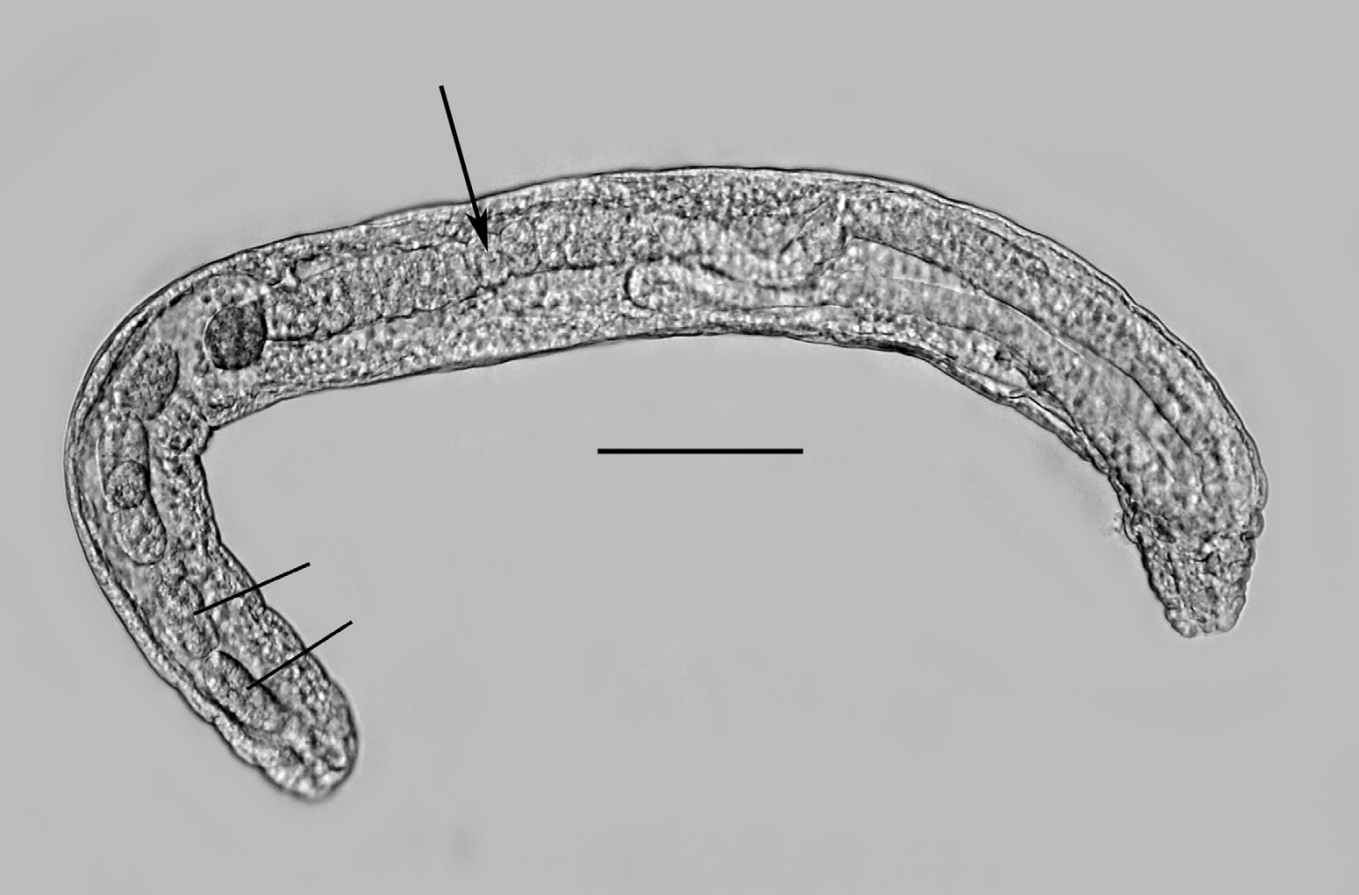
**Young parasitic female of *Halophilanema prolata***. Arrow shows developing oocytes. Lower lines show sperm packets. Bar = 108 μm.

**Figure 15:**
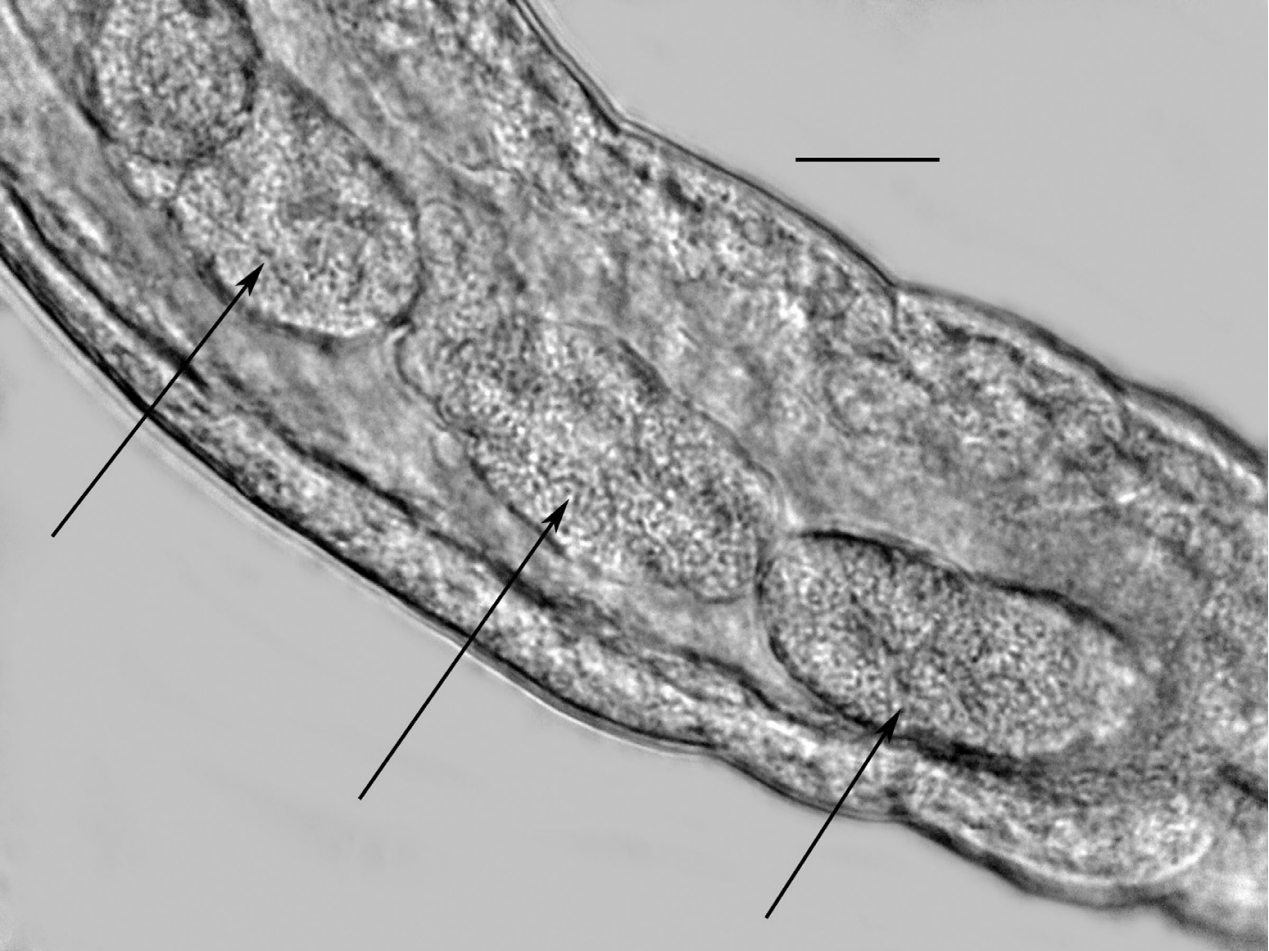
**Three sperm packets (arrows) in developing parasitic female of *Halophilanema prolata *shown in Figure 13**. Bar = 17 μm.

Mature parasitic female. (Figures 10, 11, 12, 13, 14, 15) (n = 10): length, 3.1 (2.1-3.9) mm; greatest width, 236 (151-284); stylet length, 13 (11-17); head to excretory pore, 256 (245-271); head to tip of ovary, 147 (10-507); % vulva, 97 (96-99). Body grey-tan, tubular, curved dorsally when young, but becoming flaccid with irregular elongate shape in age; ovary reflexed 2-4 times; vulva subterminal, often positioned on short prominence; excretory pore and anus faint; nerve ring obscured; ovoviviparous; sperm in uterus of young parasitic females arranged in series of packets ranging from 30-60 in length and 15-25 in width.

**Figure 16:**
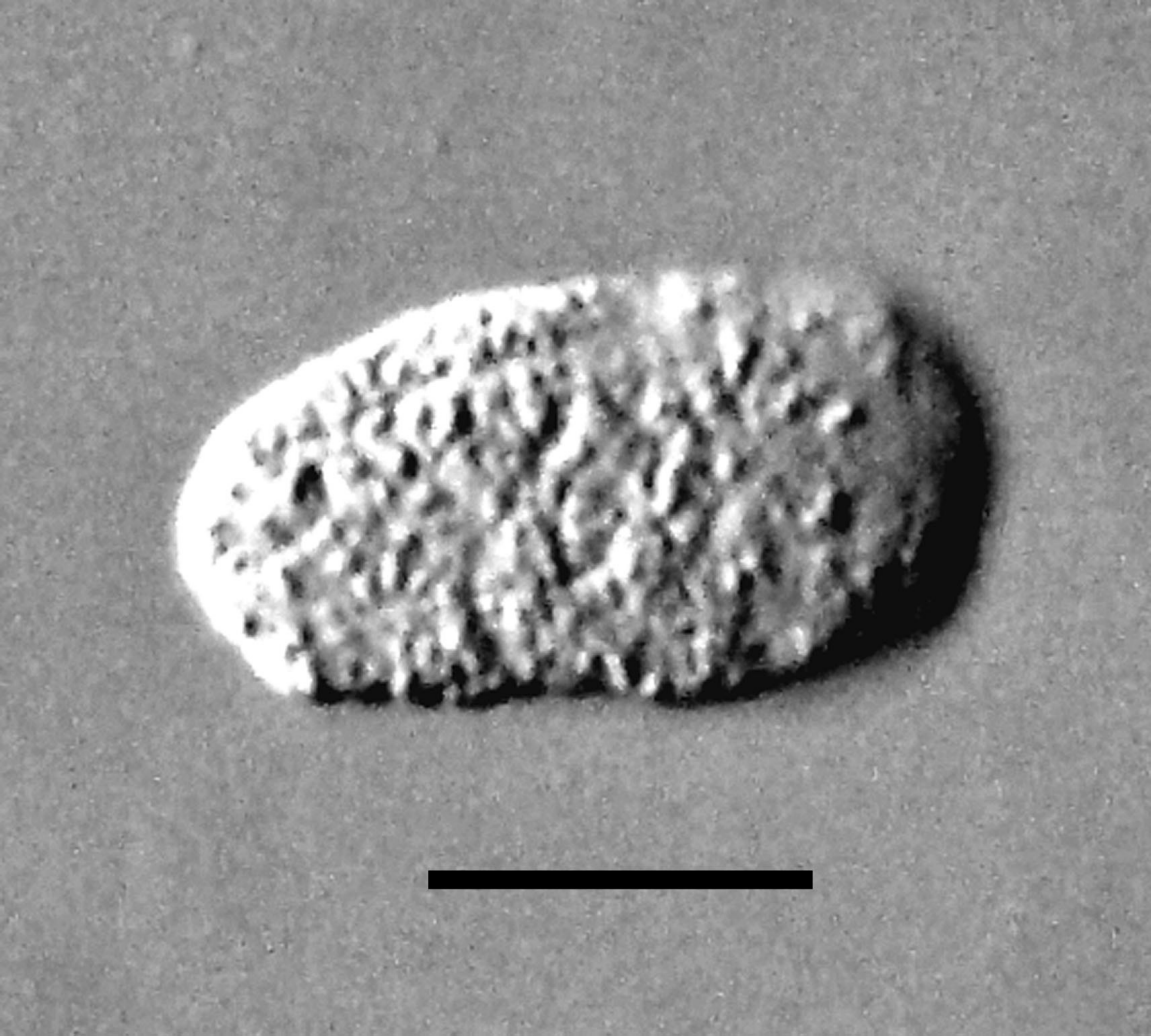
**Egg of *Halophilanema prolata *from uterus of parasitic female**. Bar = 46 μm.

**Figure 17:**
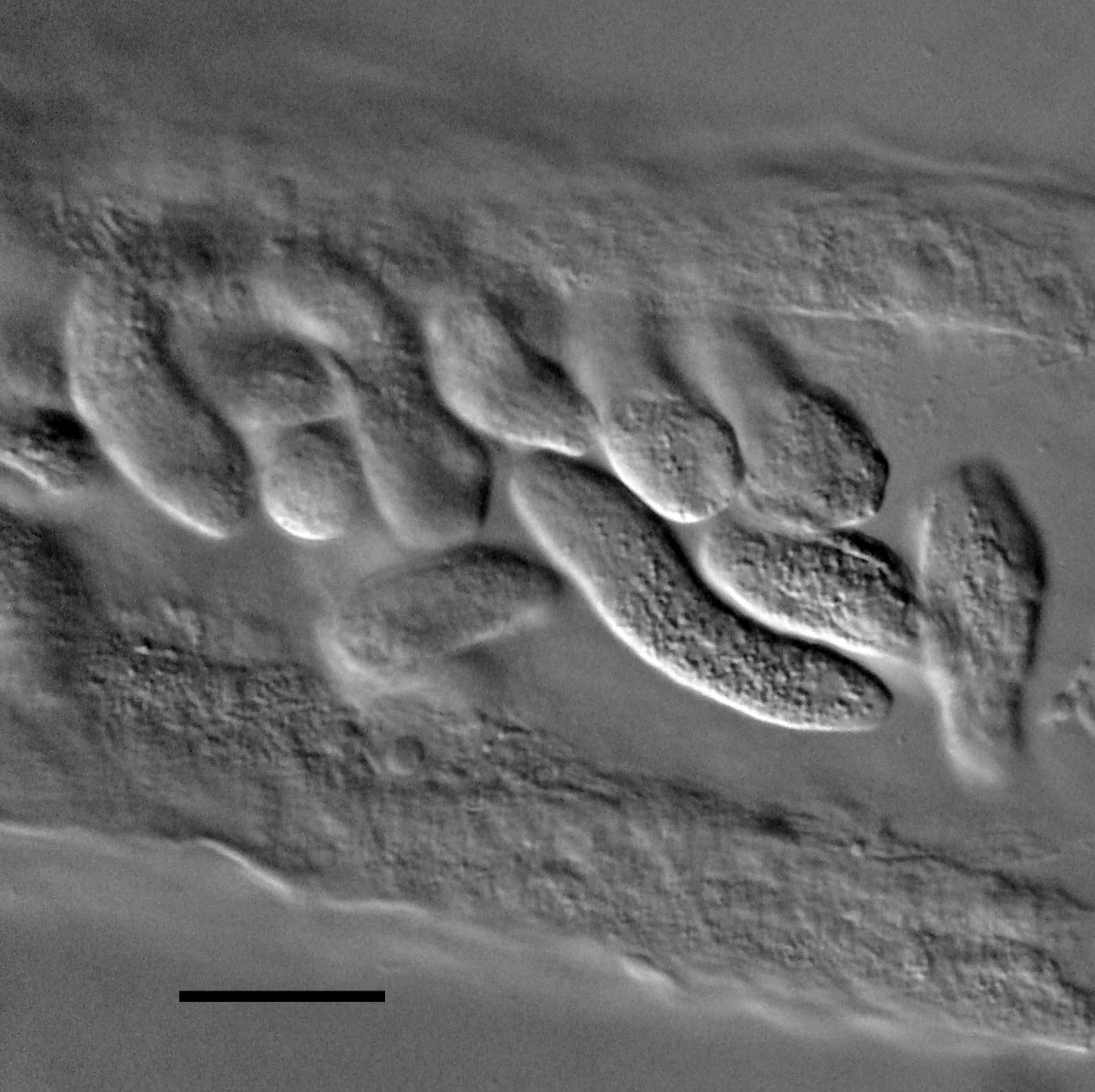
**Developing embryos of *Halophilanema prolata *in uterus of parasitic female**. Bar = 42 μm.

**Figure 18:**
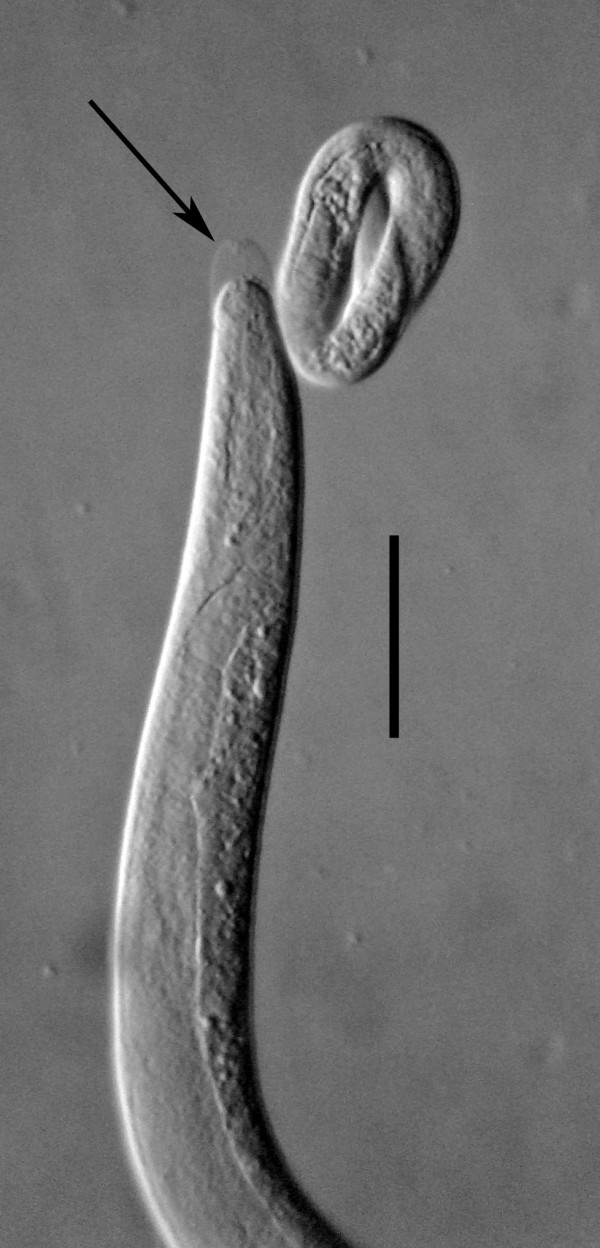
**Coiled first stage juvenile within egg membrane and molting 2^rd ^stage juvenile of *Halophilanema prolata *from uterus of parasitic female**. Arrow shows partially shed second stage cuticle. Bar = 41 μm.

**Figure 19:**
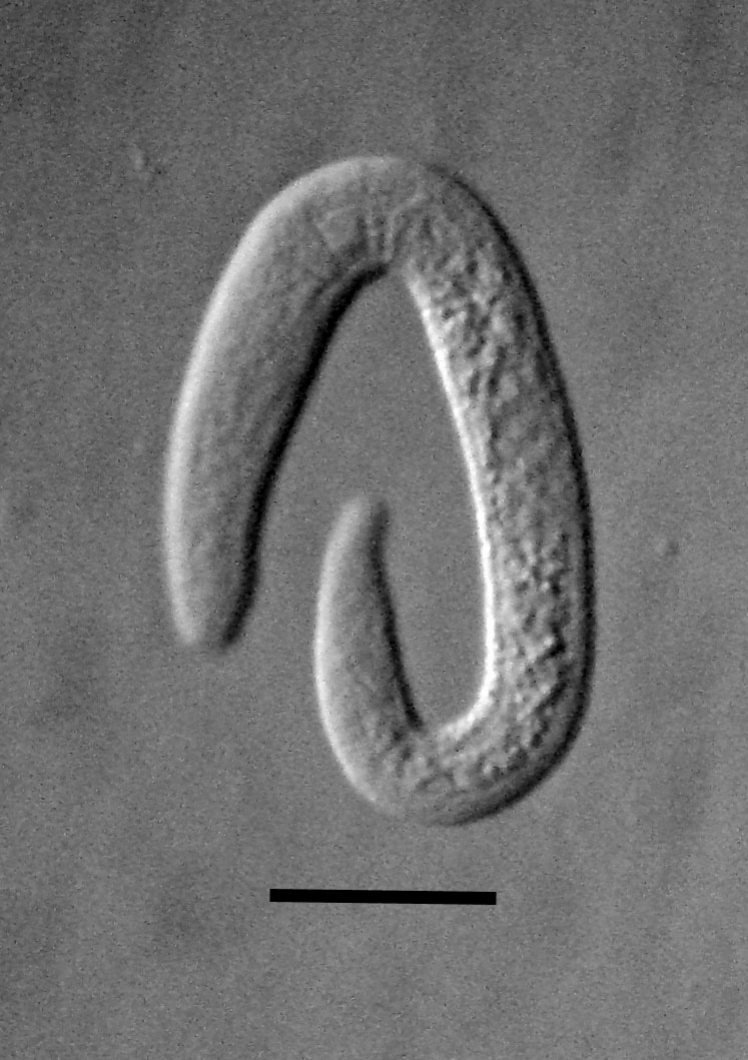
**Second stage juvenile of *Halophilanema prolata *in uterus of parasitic female**. Bar = 26 μm.

**Figure 20:**
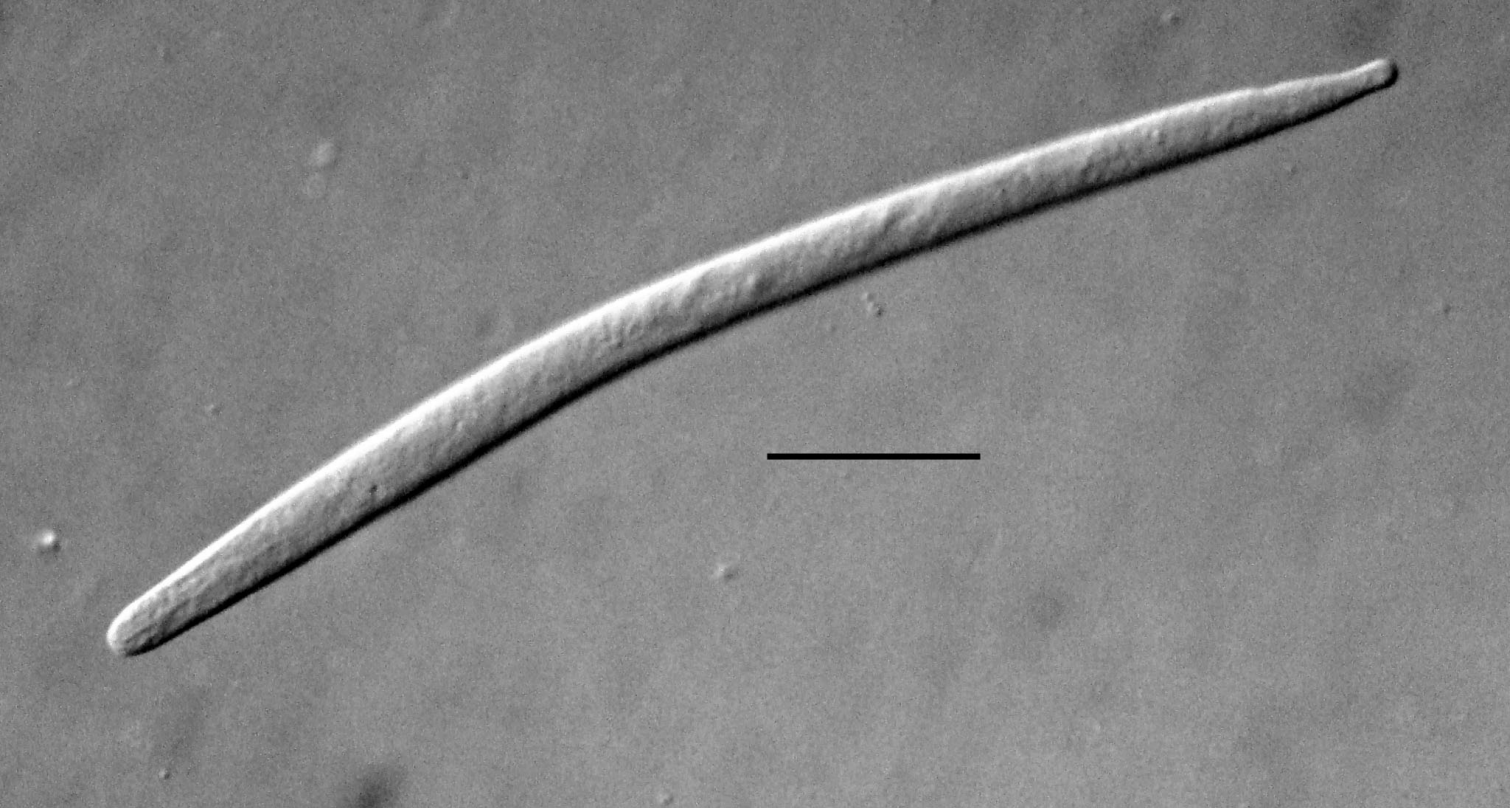
**Early third stage juvenile of *Halophilanema prolata *from uterus of parasitic female**. Bar = 42 μm.

**Figure 21:**
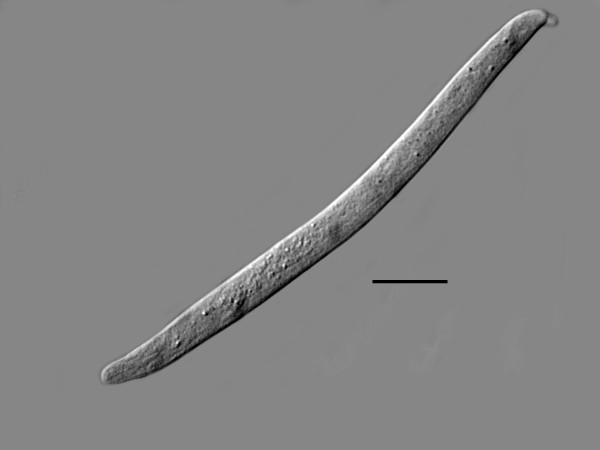
**Developing third stage juvenile of *Halophilanema prolata *from uterus of parasitic female**. Bar = 42 μm.

**Figure 22:**
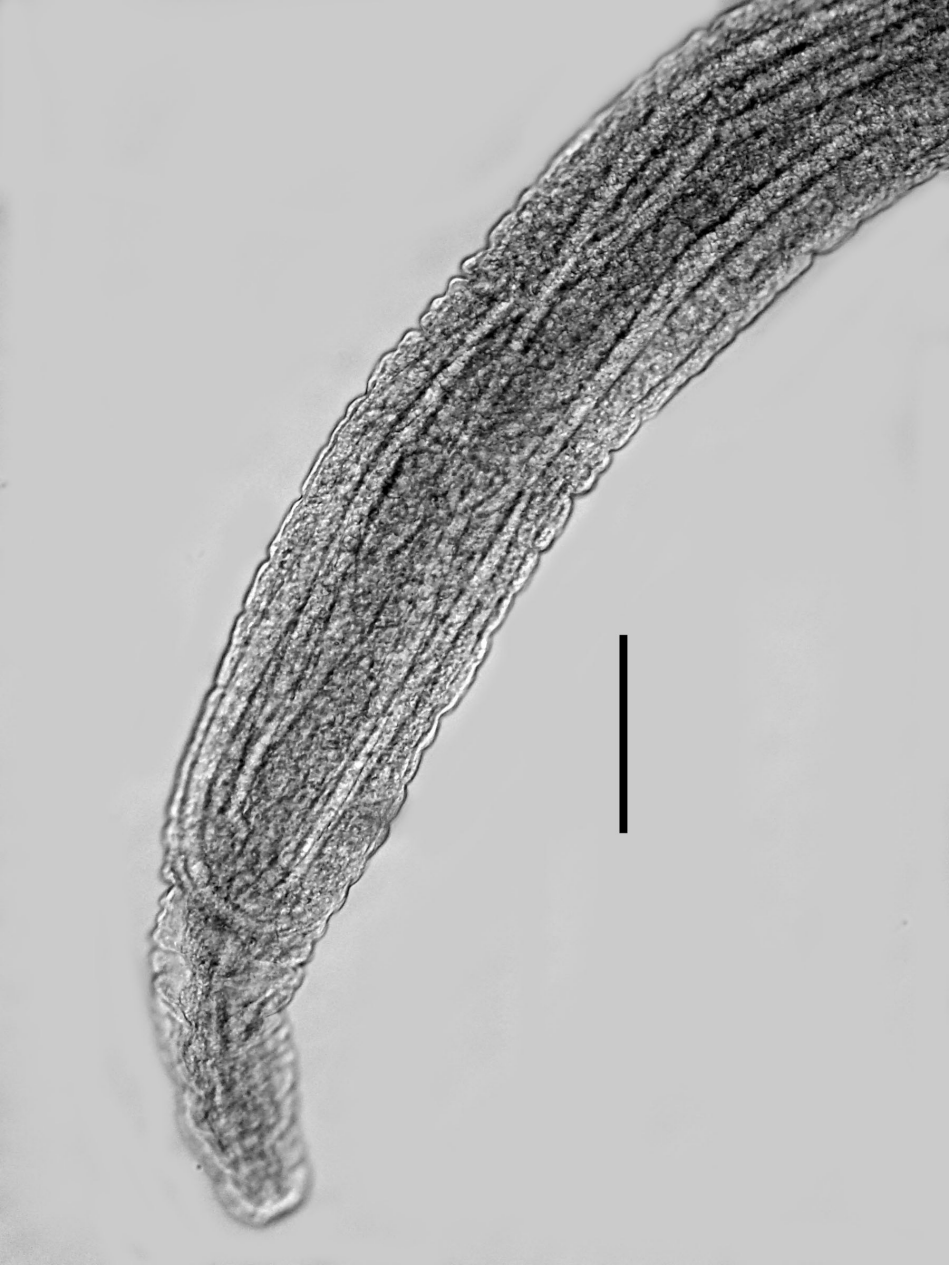
**Mature third stage juveniles in uterus of parasitic female of *Halophilanema prolata***. Bar = 120 μm.

*Immature stages*. (Figures 16, 17, 18, 19, 20, 21, 22): Length of eggs, 38 (32-48); width of eggs, 29 (19-38); first stage juveniles range from 60-115 in length, second stage juveniles range from 174-222 in length; third stage juveniles range from 260-400 in length; juveniles molt twice in uterus of mature parasitic females to reach third stage (Figures 20, 21, 22); fourth stage juveniles range from 500-580 in length.

Host: The parasites occur in the body cavity of immature and adult *Saldula laticollis *(Reuter)(Hemiptera: Saldidae). The adult hosts range in size from 4.0-5.0 mm.

Etymology: The generic epithet is taken from the Greek "halos" = sea salt, the Greek "philia" = fondness, and the Greek "nema" = thread. The specific epithet is taken from the Latin "prolata" for elongated, in reference to the long, slender free-living stages (Figure 4).

Holotype: Free-living male (USDANL # T-661t) and paratype (free-living female- USDANL # T-6122p) deposited in the USDA Nematode Laboratory, Beltsville, Maryland. Paratypes deposited in the author's collection.

Locality: Waldport (Lane County), Oregon.

## Conclusions

During organogenesis of *H. prolata*, the embryos (Figure 16) first elongate (Figure 17) then undergo a 2-fold and 3-fold stage in the females' uterus (Figures 18, 19). If not for a very fine membrane enclosing the embryo, *Halophilanema *would be considered viviparous rather than ovoviviparous. The nematode juveniles pass their second and third stages in the uterus of the parasitic female (Figures 20, 21, 22) and emerge into the hemocoel of the host as mature third stage juveniles. These stages leave the host, molt and mate in the immediate environment.

Spicular ribs are rare in the Allantonematidae [4,5] and while a single horizontal rib was depicted on the spicule of *Howardula oscinellae *(Goodey)[6], the paired horizontal ribs on the spicule lamina of *Halophilanema *appear to be a unique character.

Marine nematodes living permanently in the oceans are isotonic with sea water and experience little or no osmotic gradients [7], however *Halophilanema *is essentially a terrestrial nematode that has entered a marine environment. It is evident that *H. prolata *has acquired a wide tolerance to osmotic and ionic stress from living in the intertidal zone. The free-living stages of *Halophilanema *must have a low cuticular permeability constant, since when they were placed directly in 70% alcohol or 5% formalin at 20°C, they remained active for 48 hours. These stages probably excrete salt through their gut, excretory system and anisotropic cuticle, similar to marine nematodes. A corresponding tolerance to osmotic change occurs with the intertidal rhabditid, *Pellioditis marina *(Bastian, 1865), which has a terrestrial origin [8].

Infection rates of *S. laticollis *ranged from 0% to 85% and were highest in the tinted sand area of the *Juncus *zone (Figure 1). Outside this area, the infection rate was much lower. Two weeks later (October 17, 2001), after the fall rains and cold weather arrived, populations of *S. laticollis *dropped off suddenly and none of the few remaining adult bugs (N = 20) in the tinted sand area were parasitized. Previous studies showed that *S. laticollis *hibernated from December to April [3] but the initiation of hibernation may vary from year to year. While some free-living stages of *Halophilanema *were found in the yellow-tinted sand, they would have to infect the remaining population of bugs before being washed away by wave action during winter storms.

It is not known at what host stage infection occurs during the year, since only last stage nymphs and adults were present at this late date. Based on life cycle studies of *S. laticollis *along the Oregon coast, there is a spring generation and two summer generations. The infected bugs collected on October 3 would correspond to the end of the second summer generation [3]. The life cycle of the bugs is relatively short during the summer months with egg hatch to adult death ranging between 63 and 192 days (at 18°C). It is possible that all three host generations are infected with *Halophilanema*. The nematodes probably overwinter inside the bugs and emerge in the spring during host oviposition. Since the reproductive organs of parasitized bugs were atrophied, there may be a mock oviposition where infected bugs expel nematodes instead of eggs.

This is the first description of a nematode infecting an intertidal insect and the first complete description of an allantonematid parasite of a hemipteran. The only previously known allantonematid parasites of hemipterans occurred in fresh water hosts of the families Veliidae, Nepididae and Gerridae [9]. Poisson [9] described three separate nematode species in the genus *Bradynema *zur Strassen from each of the above hosts. While they are clearly members of the Allantonematidae, their placement in *Bradynema *is questionable. With the exception of a molting male of *B. veliae *Poisson, only the parasitic females of these three species were described and it is not possible to obtain a generic placement without free-living adults of both sexes. The molting juvenile male of *B. veliae *Poisson has long, narrow spicules and a slender tail. While these characters separate this species from *Halophilanema*, it is not typical for members of the genus *Bradynema*. The parasitic females of *B. nepae *and *B. gerridis *have the vulva positioned more anteriorly than that of *Halophilanema*. Whereas none of these species are similar to *Halophilanema*, they are the only records of allantonematids parasitizing freshwater hosts.

Within the Allantonematidae, there is a range of stylet development in the males. They can lack a stylet and gland openings completely or posses a well developed stylet with gland openings as in *Halophilanema*. Males have no use of a stylet or pharyngeal glands since they never enter a host. Nor do the third stage juvenile males (or third stage juvenile females for that matter) have a stylet strong enough to assist exiting the host. It is possible that the degree of male stylet development is correlated with the evolutionary period that the particular nematode clade has parasitized its host. Generic lineages containing males that have lost their stylets, such as *Bradynema *and *Howardula *Cobb, could have had a longer period of parasitism than clades like *Halophilanema *with males containing well-developed stylets. In other words, the presence of a male with a well-defined stylet with dorsal and ventral pharyngeal gland openings and atrophied glands as in *Halophilonema *indicates a fairly short period of parasitism, evolutionarily speaking, *Halophilanema *probably was a free-living intertidal or shore nematode that fed on microorganisms, especially fungi, in the intertidal habitat before becoming an insect parasite. This scenario implies that *Halophilanema *became parasitic when saldids entered the intertidal environment. This infers that the Allantonematidae is a polyphyletic group with different lineages adapting parasitism at different time periods. The earliest known allantonematid lineage occurs in Eocene Baltic amber [10].

There are also reports of mermithid nematodes infecting saldids [11]. Mermithids were stated to parasitize the European *Saldula pallipes *Fabricius and *Saldula saltatoria *(L.), as well the North American *Saldula laticollis *[3]. The latter discovery was in the same general area of the present study. Unfortunately the "mermithid" specimens mentioned by Stock and Lattin [3] were never described, figured or preserved. No evidence of mermithids was obtained during the present study and it is possible that the nematodes observed by Stock & Lattin [3] were developing parasitic females of *Halophilanema*, since the latter can reach up to nearly 4 mm in length and superficially resemble mermithid nematodes (Figure 2).

**Figure 23:**
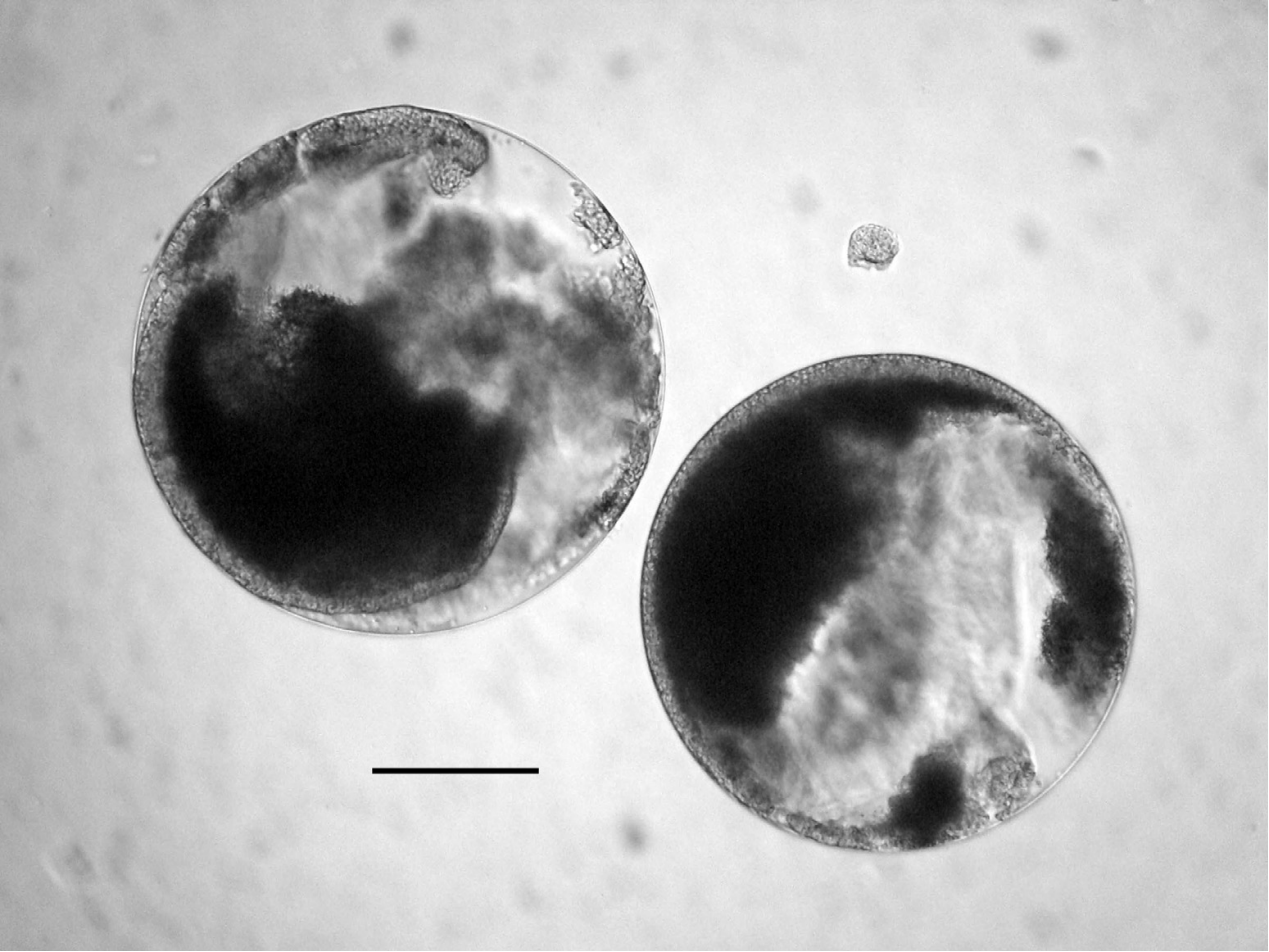
**Two gametocysts of a eugregarine parasite from the body cavity of *Saldula laticollis***. Bar = 113 μm.

During the present study, cysts of a eugregarine parasite were encountered inside the body cavity of *S. laticollis *(Figure 23). These cysts ranged in diameter from 252-347 μm and contained navicular spores ranging from 7.0 to 8.6 μm in length. The fat body and reproductive organs of infected individuals were greatly reduced. This is the first record of eugregarines in saldid bugs.

In accordance with section 8.6 of the ICZN's International Code of Zoological Nomenclature, copies of this article are deposited at the following five publicly accessible libraries: Natural History Museum, London, UK; American Museum of Natural History, New York, USA; Museum National d'Histoire Naturelle, Paris, France; Russian Academy of Sciences, Moscow, Russia; Academia Sinica, Taipei, Taiwan.

## Competing interests

The author declares that they have no competing interests.

## Authors' contributions

GP discovered the nematode, wrote the paper and supplied the figures.
